# A ready-to-use machine learning tool for symmetric multi-modality registration of brain MRI

**DOI:** 10.1038/s41598-023-33781-0

**Published:** 2023-04-24

**Authors:** Juan Eugenio Iglesias

**Affiliations:** 1 Athinoula A. Martinos Center for Biomedical Imaging, Massachusetts General Hospital and Harvard Medical School, Boston, 02129 USA; 2 Department of Medical Physics and Biomedical Engineering, University College London, London, WC1V 6LJ UK; 3 Computer Science and Artificial Intelligence Laboratory, Massachusetts Institute of Technology, Boston, 02139 USA

**Keywords:** Software, Biomedical engineering

## Abstract

Volumetric registration of brain MRI is routinely used in human neuroimaging, e.g., to align different MRI modalities, to measure change in longitudinal analysis, to map an individual to a template, or in registration-based segmentation. Classical registration techniques based on numerical optimization have been very successful in this domain, and are implemented in widespread software suites like ANTs, Elastix, NiftyReg, or DARTEL. Over the last 7–8 years, learning-based techniques have emerged, which have a number of advantages like high computational efficiency, potential for higher accuracy, easy integration of supervision, and the ability to be part of a meta-architectures. However, their adoption in neuroimaging pipelines has so far been almost inexistent. Reasons include: lack of robustness to changes in MRI modality and resolution; lack of robust affine registration modules; lack of (guaranteed) symmetry; and, at a more practical level, the requirement of deep learning expertise that may be lacking at neuroimaging research sites. Here, we present *EasyReg*, an open-source, learning-based registration tool that can be easily used from the command line without any deep learning expertise or specific hardware. *EasyReg* combines the features of classical registration tools, the capabilities of modern deep learning methods, and the robustness to changes in MRI modality and resolution provided by our recent work in domain randomization. As a result, *EasyReg* is: fast; symmetric; diffeomorphic (and thus invertible); agnostic to MRI modality and resolution; compatible with affine and nonlinear registration; and does not require any preprocessing or parameter tuning. We present results on challenging registration tasks, showing that *EasyReg* is as accurate as classical methods when registering 1 mm isotropic scans within MRI modality, but much more accurate across modalities and resolutions. *EasyReg* is publicly available as part of FreeSurfer; see https://surfer.nmr.mgh.harvard.edu/fswiki/EasyReg.

## Introduction

### Background

Image registration is the problem of finding a spatial correspondence between two or more images, i.e., finding a spatial transform that aligns them. Registration of 2D images was initially driven by the computer vision community, with the objective to align photographs of the same scene acquired at different times, viewpoints, or sensors^1^. Soon after, the medical imaging community started building on these tools to develop a huge body of methods that adapted to the peculiarities of medical images, such as the anisotropic resolution, modality-specific artifacts, or the higher dimensionality of the images. The great majority of these techniques were based on numerical optimization of an objective function estimating the similarity between the reference and deformed images—where the latter is a function of the deformation parameters that one optimizes^2^. Throughout the rest of this article, we refer to these methods as “classical methods”—as opposed to modern learning-based methods.

In human neuroimaging with brain MRI, medical image registration has been widely used. Barring large age gaps or presence of pathology that greatly distorts brain anatomy (e.g., tumors, very strong atrophy), volumetric registration methods generally succeed at aligning subcortical brain structures; we note that surface methods are instead used for the cortex, due to its convoluted geometry^3^. There is an abundance of publicly available methods that can accurately register brain MRI scans of the same contrast at isotropic resolution, such as those implemented in packages like ANTs^4^, NiftyReg^5^, Elastix^6^, or DARTEL^7^. These methods are used routinely in neuroimaging, e.g., in radiation therapy planning^8^, registration-based segmentation^9^, longitudinal analysis^10^, estimating change across timepoints^11^, or to bring subjects to a common coordinate frame for statistical analysis^12^.

Classical registration methods build on well-established mathematical tools like numerical optimization^13,14^ or Lie algebra^7,15^, which equip them with properties that are desirable when spatially mapping brain images. For example, smooth and invertible deformation fields that preserve topology can be obtained with diffeomorphic models like the Log-Euclidean framework^15^ or large deformation diffeomorphic metric mapping (LDDMM^16^). Symmetric frameworks measure image similarity in both registration directions or in a mid space while enforcing inverse consistency^17^, thus ensuring that switching the order of the inputs produces the inverse transform as a result. General-purpose objective functions can deal with artifacts or differences in intensity profiles; for example, the local normalized cross correlation function is robust against bias fields^4^, and information theory metrics like mutual information can map intensities across modalities^18^.

Learning-based registration methods emerged in the mid 2010s as part of the deep learning revolution. Early learning-based methods used direct voxel-wise supervision on the deformation fields, with ground truth obtained synthetically^19^ or with classical methods^20^. These methods were superseded by unsupervised approaches using objective functions similar to those used by classical approaches^21,22^. These unsupervised methods yield higher performance because they avoid overfitting to ground truth fields in flat image regions. Learning-based methods are not only much faster than classical approaches (one or two orders of magnitude faster), but also compatible with features that classical methods do not support. For example, they can be supervised with landmarks or volumetric segmentations, to achieve higher accuracy than classical methods^23^. This supervision can also be used to train neural networks that register images of different contrast much more accurately than mutual information^24^. Another advantage of learning-based registration is that it can be used as a building block for more complex-meta architectures. For example, we used it to guide contrast synthesis between MRI and histology^25^.

Despite all its potential advantages, the adoption of learning-based registration in neuroimaging pipelines has so far been nearly inexistent. We believe that there are several reasons contributing to this lack of adoption. First, the lack of robustness to changes in contrast and resolution. Convolutional neural networks (CNNs) used in registration are generally fragile to changes in MRI modality, even with aggressive augmentation^26^. Another reason is the lack of robust affine modules in registration architectures. While affine registration layers do exist^21^, their robustness has not been widely demonstrated in publicly available software. Other minor reasons are the lack of symmetry and inverse consistency in most learning-based approaches, as well as the lack of publicly available implementations that do not require expertise in deep learning or complex pipelines—which precludes routine use at many neuroimaging research sites.

### Contribution

In this article, we present *EasyReg*, a deep learning registration tool for brain MRI scans that combines the features of classical registration methods with the speed and other advantages of learning-based methods, while being straightforward to use from the command line. Specifically our new method:

- Capitalizes on our recent work on domain randomization (SynthMorph^24^) to be agnostic to MRI modality and resolution, i.e., it can register two images with different orientations (coronal, axial, sagittal), slice spacings, and pulse sequences; the deformation fields are always computed at 1 mm isotropic resolution, independently of the resolutions of the input images. We note that *EasyReg* is, along with SynthMorph, the only deep learning method that can register brain scans of any MRI contrast “out of the box”, without retraining—since CNNs trained with mutual information on real scans (e.g., T1- and T2-weighted) will not generalize to other modalities (e.g., proton density and FLAIR) without retraining.
- Combines with our recent domain-agnostic segmentation and parcellation CNN^27^ to analytically compute an affine transform to atlas space using the centroids of the parcels, while skull stripping the scans.
- Parameterizes the deformation as a stationary velocity field (SVF^15^), which provides diffeomorphic fields that preserve topology and can be easily inverted.
- Guarantees symmetry and inverse consistency by construction.
- Has no parameters to tune.
- Runs in about a minute on a modern CPU.
- Once FreeSurfer has been installed, *EasyReg* can be used directly from the command line, without the need to set up virtual environments, install dependencies, or preprocess the scans (e.g., skull stripping, or linear registration to a reference space are not needed).

### Further related work

Even though they only capture a limited spectrum of diffeomorphisms, SVFs^15^ have been very popular in classical image registration^7,28^ thanks to their computational efficiency: an SVF can be quickly integrated into a (diffeomorphic) deformation field with the scale and square algorithm^29^. The SVF is typically non-parametric, but can also be parameterized, e.g., with B-splines^30^. While the inverse field can be easily computed simply by integrating the negated SVF, symmetry is not guaranteed by the sole use of SVFs. Symmetric algorithms can be obtained by computing the similarity metric in the space of both input images or at the midpoint of the flow between them^31^. An example of the latter is the widespread ANTs package^4^.

Inter-modality registration of brain MRI relies almost exclusively on information theory metrics like mutual information^18^—even though correlation metrics can sometimes be used if the relationship of the intensities of the two modalities is approximately linear. While mutual information has been very successful in rigid and affine registration, its excessive flexibility makes its application to inter-modality registration ill posed^32^. Moreover, possible differences in resolution between the two input scans are (to the best of our knowledge) generally disregarded.

Learning-based registration methods have successfully implemented diffeomorphic models by integrating SVFs with vector field exponentiation layers^22,33^—which can be achieved by unrolling the scale and square algorithm. Differences in MRI modality and resolution can be effectively handled with supervision, leading to higher accuracy than classical methods^24^. This is achieved by training neural networks with losses that exploit additional high-resolution images or anatomical labels that are available during training but *not* as test time. This way, the network can effectively learn the features that best align the underlying labels (or high-resolution images) without having access to them^22^.

Since symmetry can reduce systematic bias caused by the order of input images and increase robustness, learning-based registration approaches have sought to incorporate this features. A number of works have attempted to promote symmetry by adding inverse-consistency losses^34–41^. These losses are generally based on computing two deformation fields (one with the order of the images switched), composing them, and penalizing the deviation from identity. Some works also deform the images twice with both fields and penalize deviations from the original images, to further encourage cycle consistency^42,43^. However, these approaches do not guarantee inverse consistency at test time, when a new pair of images is fed to the neural network.

Affine registration can be implemented as a regression layer in learning-based approaches, but often fails when the inputs are not already roughly aligned^21^. An alternative to regression is to pose affine registration as a keypoint matching problem. This approach is common in the classical computer vision literature (best represented by the SIFT algorithm^44^), which has evolved from matching handcrafted features to capitalizing on features obtained with deep learning approaches^45^. A first application of this keypoint matching to brain MRI was proposed in^46^. Rather than keypoints, our proposed approach builds on the idea of aligning segmentations, which is and old idea in the classical registration literature (see, e.g.,^47^) and also been explored in the deep learning registration literature (e.g.,^48^). To the best of our knowledge, no affine registration method for brain MRI is publicly available.

**Figure 1:**
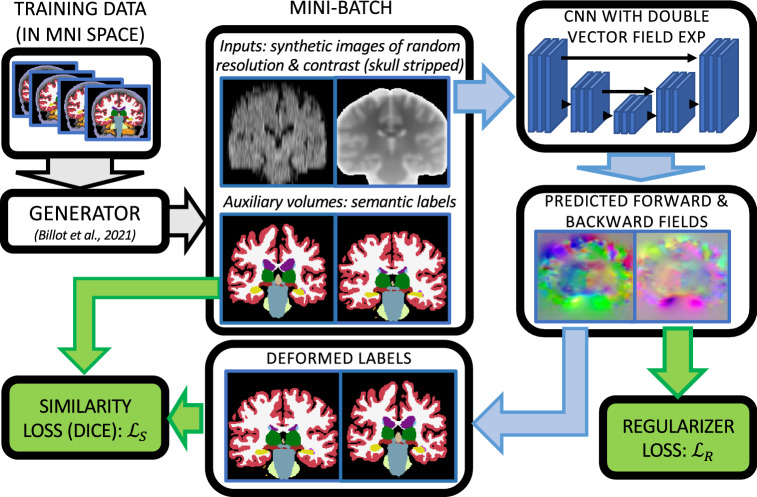
Overview of the training procedure used by *EasyReg*. The gray arrows follow the generator; the blue arrows follow the layers of the neural network; and the green blocks represent the different terms of the loss. We emphasize that the segmentations are used to compute the loss, but are not given as input to the CNN.

## Methods

### Preliminaries

Pair-wise registration involves two images: fixed (also known as reference or target) and moving (also know as floating or source). The goal is to find a geometric transform that maximizes the similarity of the fixed image and the deformed moving image. The transform is usually parameterized by a vector of parameters $\varvec{p}$, and registration posed as an optimization problem:

1 $$\begin{aligned} {\hat{p}} = \mathop {\text{ argmax }}\limits _{\varvec{p}} {\mathscr {S}}{[}F(\varvec{x}), M(\varvec{T}(\varvec{x}; \varvec{p})){]} + {\mathscr {R}} {[}\varvec{T}(\varvec{x}; \varvec{p}){]}, \end{aligned}$$

where $\varvec{x}$ is the spatial location, *F* is the fixed image, *M* is the moving image, $\varvec{T}$ is the transform, ${\mathscr {S}}$ is a function that measures the similarity between the fixed and deformed images, and ${\mathscr {R}}(\varvec{T})$ is a regularizer that encourages the regularity of the transform. Equation (1) is typically solved with a numerical optimizer, e.g., gradient ascent.

In learning-based registration, ones instead predicts the transform directly with a neural network parameterized by its own vector of parameters $\varvec{\theta }$ (the weights of the network):

$$\begin{aligned} \hat{\varvec{T}}(\varvec{x}) = \varvec{h}[F(\varvec{x}), M(\varvec{x}); \varvec{\theta }]. \end{aligned}$$

The parameters of the network $\varvec{\theta }$ are optimized during training, and are fixed at test time. The loss ${\mathscr {L}}$ used during training depends on the type of approach. In unsupervised learning paradigms, losses are similar to those used classical registration, i.e., Eq. (1):

$$\begin{aligned} {\mathscr {L}} = \ {\mathscr {S}}(F(\varvec{x}), M(\varvec{x}) \circ \varvec{h}[F(\varvec{x}), M(\varvec{x}); \varvec{\theta }] ) + {\mathscr {R}} (\varvec{h}[F(\varvec{x}), M(\varvec{x}); \varvec{\theta }]), \end{aligned}$$

which can be optimized with respect to $\varvec{\theta }$ using stochastic gradient descent approaches on an unlabeled training dataset: at every mini-batch, one just samples two random scans from the training data, computes the loss, and backpropagates through the neural network to update its weights $\varvec{\theta }$.

### Overview of algorithms

**Figure 2:**
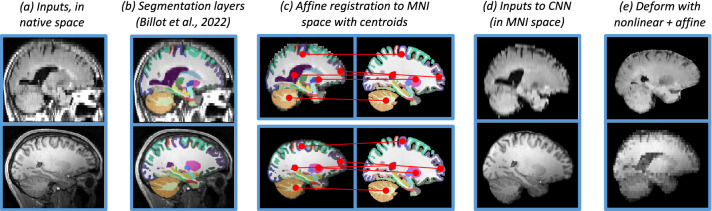
Overview of the inference procedure used by *EasyReg*, using a 5 mm axial FLAIR scan and a 1 mm isotropic MPRAGE as sample inputs. (**a**) Sagittal slices of the input scans. (**b**) Output of segmentation layers from^27^, which include subcortical structures and cortical parcels. (**c**) The centroids of the different labels are used to compute an affine transform to MNI space using least squares regression; the centroids of the MNI atlas are precomputed. (**d**) The transforms are used to bring the two (skull-stripped) scans into the voxel space of the MNI reference, which is the space where the CNN was trained. (**e**) The CNN is used to compute the forward and backward deformation fields, which are composed with the affine transforms in order to obtain the final fields and finally deform the original images.

Our method is summarized in Figs. 1 and 2, which display the pipelines used for training the CNN and testing on a pair of input scans, respectively.

Training relies on a large dataset of 3D segmentations of brain structures, which are affinely pre-aligned to a reference space with fixed orientation and voxel dimensions; we used the MNI template^49^ with identity voxel-to-world coordinate transform matrix. Working in MNI space has two advantages. First, affine registration (for which robust learning methods do not exist) does not need to be modeled by the CNN; instead, we handle it with a geometric module based on centroids of brain structures (see below). And second, the CNN only needs to learn to register images with a consistent orientation, which makes the learning procedure easier as the CNN does not need to learn to estimate pose.

At each training iteration, a mini-batch consisting of two segmentations is drawn from the training dataset. These segmentations are used to synthesize two skull-stripped scans with random contrast and resolution. The two synthetic images are fed to a CNN that estimates a stationary velocity field in a symmetric fashion (i.e., switching the orders returns the negated field). This SVF and its negated version are integrated in order to obtain the forward and backward deformation fields, which are used to warp the labels. The training loss consists of a similarity term and a regularizer. The former is the Dice overlap between the original and deformed labels, in both fixed an moving image space; the latter penalizes the irregularity of the deformation field—specifically, the square of the magnitude of its spatial gradient.

At test time, the input scans (Fig. 2a) go through a segmentation and parcellation layer that is also trained with synthetic data to be agnostic to MRI modality and resolution^27^ (Fig. 2b). The centroids of the segmented structures are then used to compute an affine registration to MNI space, which is estimated with a simple least squares fit; the centroids for the MNI atlas are precomputed (Fig. 2c). We note that this step is differentiable, so it enables the use of the whole architecture in training of more complex meta-architectures. The input scans are then skull stripped with the union of the segmentation labels and linearly deformed to our reference space (Fig. 2d). This deformation resamples the images to a 1 mm isotropic grid, irrespective of their original resolution. At that point, the resampled images can be fed to the trained CNN, which expects 1 mm isotropic inputs, and produces the forward and backward nonlinear fields (again, at 1 mm resolution). These fields are finally concatenated with the affine transforms to obtain the final forward and backward transforms between the two native spaces.

### Training

#### Training dataset

To train *EasyReg*, we need a large pool of 3D segmentations affinely registered to a reference space. Crucially, these segmentations do not need to be manual: since synthetic images will be generated from them, the alignment between images and labels will be perfect by construction. In practice, we obtained the training data as follows.

First, we defined the reference space. We used the symmetric version of 2009 MNI template^49^ at 1 mm resolution, which we reoriented and shifted such that the voxel-to-world transform matrix was the identity matrix. We processed this template with SynthSeg^26^ in order to obtain segmentations for 97 regions of interest (ROIs): 68 cortical and 29 subcortical. The centroids of these ROIs were computed and saved for later use during inference. SynthSeg also provided an intracranial mask, which was used to skull strip the template.

Next, we compiled a set of 1000 1 mm isotropic MPRAGE scans from two publicly available datasets: 500 randomly selected from ADNI^50^ and 500 randomly selected from HCP^51^. These two datasets complement each other well; ADNI mostly comprises elderly subjects (including controls and cases with strong Alzheimer-related atrophy and white matter lesions), whereas HCP consists of younger controls. These scans were skull stripped with SynthStrip^52^ and affinely registered to the stripped MNI template with a block matching approach implemented in NiftyReg^53^. Finally, the registered scans were segmented with SynthSeg, without subdividing the cortex into parcels - since parcels are not needed to synthesize the gray matter and are very difficult to register volumetrically.

We note that:

- We register and segment, in that order (i.e., as opposed to segmenting in native space and deforming the segmentation), in order to avoid resampling segmentations with nearest neighbors.
- The described preprocessing is only needed for the training dataset; running SynthStrip or registering with NiftyReg is not required at test time (see Section “Registration” below).
- Our pool of 1000 cases yields almost half a million unique pairs of 3D segmentations for training.

#### Synthetic data generator

In order to make the neural network agnostic to the orientation (axial, coronal, sagittal), MRI modality, and resolution of the inputs (slice thickness, slice spacing), we adopt a domain randomization approach that we have successfully used in other brain MRI analysis problems (e.g.,^26,54^). The key idea is that, by randomizing the orientation, resolution, and MR contrast at every iteration during training, the CNN learns features that are agnostic to MR contrast and resolution. The fine details of the synthetic data generator can be found in^55^, but we summarize them here for completeness.

Given a 3D segmentation at 1 mm isotropic resolution, we obtain a corresponding image of random contrast and resolution as follows. First, we group the labels with similar tissue types. For example, the different cerebrospinal fluid (CSF) structures are grouped into a single CSF label. Next, we sample a mean and variance for each label from uniform distributions. These means and variances are used to produce a first “Gaussian image” at 1 mm isotropic resolution. This Gaussian image is a sample of a Gaussian mixture model conditioned on the underlying segmentation. The Gaussian image is further corrupted by a random, synthetic, multiplicative bias field. This field is obtained by sampling a small, zero-mean Gaussian field (4 × 4 × 4 voxels), taking voxel-wise exponentials, and upscaling to the full voxel size of the Gaussian image.

After randomly choosing an orientation (axial, sagittal, coronal), a slice thickness (sampled from a uniform distribution) is simulated with a one-dimensional Gaussian kernel in the perpendicular direction of the orientation. Next, a slice spacing (also sampled from a uniform distribution) is simulated by downsampling the (bias-corrupted) Gaussian image in the direction perpendicular to the orientation, by a factor equal to the ratio between the sampled spacing and 1 mm. Finally, this image is min-max normalized and scaled back up to the original resolution, i.e., 1 mm isotropic. This resampling operation (which will also happen at test time, see Section “Registration”), ensures that that the size of the input to the CNN is constant and equal to the size of the underlying segmentation. This yields deformation fields that are always 1 mm isotropic and enables computation of the loss on a 1 mm grid, independently of the resolution of the input scans – which can be different for the fixed and moving images.

#### Symmetric estimation of nonlinear deformation with a CNN

In order to estimate the nonlinear deformation, we train a CNN that takes as input two scans in the voxel space of the MNI reference, and estimates a diffeomorphic deformation field and its inverse in a symmetric fashion. Let $\varvec{g}[F(\varvec{x}), M(\varvec{x}); \varvec{\theta }]$ be a set of convolutional layers with weights $\varvec{\theta }$ that estimate (regress) an SVF from two input images (which are simply concatenated in the feature dimension), and let $\varvec{V}[\cdot ]$ be a vector field exponentiation (integration) layer with no trainable parameters. Our method produces a symmetric deformation field by construction, by explicitly symmetrizing the estimate:

2 $$\begin{aligned} \hat{\varvec{v}}(\varvec{x})&= \varvec{g}[F(\varvec{x}), M(\varvec{x}); \varvec{\theta }] - \varvec{g}[M(\varvec{x}), F(\varvec{x}); \varvec{\theta }], \nonumber \\ \hat{\varvec{T}}(\varvec{x})&= \varvec{V}[\hat{\varvec{v}}(\varvec{x})], \nonumber \\ \hat{\varvec{T}^{-1}}(\varvec{x})&= \varvec{V}[-\hat{\varvec{v}}(\varvec{x})], \end{aligned}$$

where switching the order returns the inverse fields, up to the numerical precision of the vector field exponentiation^56^.

We note that:

- $\varvec{g}(F,M)$ approximates the SVF of the deformation taking *F* to the mid-space of *F* and *M*.
- Conversely, $\varvec{g}(M,F)$ approximates the SVF of the deformation taking *M* to the mid-space.
- Adding two SVFs is an approximation to the composition of the fields they parameterize (it is equivalent to truncating the Lie bracket in the Baker–Campbell–Hausdorff series, see^15,30^). Therefore, Eq. (2) approximates the SVF of the field taking *F* to *M* (i.e., *F* to mid-space, composed with mid-space to *M*).

These properties enable us to easily initialize our CNN with existing nonlinear registration CNNs based on SVFs, simply by dividing the weights and biases of the final layer by two. This is of course only an approximation due to the BCH truncation and the fact that dividing the SVF by two is not equivalent to integrating to 0.5. However, finetuning from this point is in practive much faster than training from scratch—particularly when training with domain randomized data, which is typically much slower than training with real data.

#### Training loss

Our training loss comprises a similarity term and a regularizer. As similarity term, we use the soft Dice overlap of the deformed labels^57^, computed in both fixed and moving space, for symmetry:

$$\begin{aligned} {\mathscr {L}}_S = 2 - \text {Dice}[F_{labs}, M_{labs} \circ {\varvec{T}}] - \text {Dice} [M_{labs}, F_{labs} \circ {\varvec{T}}^{-1}], \end{aligned}$$

where $F_{labs}$ is the labels (3D segmentation) of the fixed image, $M_{labs}$ is the labels of the moving image, and $\text {Dice}$ is the Dice overlap.

The regularizer is also symmetric and discourages irregularities in the deformation fields, by penalizing the magnitude of their gradients:

$$\begin{aligned} {\mathscr {L}}_R = \frac{1}{|\Omega |} \sum _{\varvec{x}\in \Omega } \left( \Vert \nabla {\varvec{T}}(\varvec{x}) \Vert ^2 + \Vert \nabla {\varvec{T}}^{-1}(\varvec{x}) \Vert ^2 \right) , \end{aligned}$$

where $\Omega$ is the image domain (i.e., $|\Omega |$ is the number of voxels). The final loss is simply the linear combination:

$$\begin{aligned} {\mathscr {L}} = {\mathscr {L}}_S + \lambda {\mathscr {L}}_R, \end{aligned}$$

where $\lambda$ is a trade-off parameter.

### Registration

#### Robust and symmetric affine registration with centroids

The CNN described above symmetrically predicts deformation fields for a pair of skull-stripped images in the voxel space of the reference (the MNI atlas). Therefore, an affine registration method is needed to bring the input scans into this reference space. Rather than relying on affine registration networks, we build on our previous CNN for segmentation and parcellation of brain scans of any MRI contrast and resolution^26,27^ (see Fig. 2b), which has three advantages: it already is agnostic to MRI modality and resolution like the rest of the method; provides the brain mask that is required by the nonlinear registration CNN; and it segments 97 ROIs that are useful not only for affine registration (see below), but potentially for other analyses as well (e.g., volumetry).

Given the soft segmentation, it is straightforward to compute the centroids of the 97 ROIs using a weighted sum, which is differentiable (Fig. 2c). Given these centroids, along with the centroids of the reference (which are precomputed and constant, as explained in Section “Training”), it is straightforward to compute the affine transform that minimizes the least squares error. If *C* is the 4 × 97 matrix with the coordinates of the centroids of the scan in homogeneous coordinates (i.e., the last row consists of ones), and $C_{ref}$ is the matrix with the centroids of the reference, the 4 × 4 affine transform matrix in homogeneous coordinates is given by:

$$\begin{aligned} {\hat{A}} = C_{ref} C^t (C C^t)^{-1}, \end{aligned}$$

which, again, is differentiable. In practice, we remove from *C* and $C_{ref}$ the rows corresponding to ROIs that have less than 100 voxels in the segmentation of either input scan, in order to make the fit robust against ROIs that are partially or totally outside the field of view of the scans.

This affine registration approach is symmetric by construction, since the two scans to be registered are independently registered to target space. The affine registration is also robust, for two reasons. First, the large number of ROIs provides 3 × 97 = 291 equations to fit 12 unknowns. And second, thanks to the cortical parcellation, many of the centroids are in the periphery of the brain, which avoids extrapolation errors that could occur in these areas if the fit was restricted to subcortical ROIs.

#### CNN inference

Once the affine transform has been computed, the input scans are resampled to the 1 mm isotropic voxel space of the MNI reference, skull stripped with the deformed segmentation, and min-max normalized (Fig. 2d). These images are then fed to the CNN that has been trained for nonlinear registration, which produces estimates of the forward and backward fields $\hat{\varvec{T}}$ and $\hat{\varvec{T}}^{-1}$ on a 1 mm isotropic grid, irrespective of the original resolution of the inputs. At that point, one can estimate the final transforms simply by concatenating the affine and nonlinear transforms—which requires interpolation of $\hat{\varvec{T}}$ and $\hat{\varvec{T}}^{-1}$, which we accomplish with a trilinear model. Finally, these transforms are used to warp the images (Fig. 2e).

In practice, we store the two final transforms in Nifti files with the same header and voxel size of the inputs (which may be different from each other), using three frames that store the real world coordinates of the location where each voxel is mapped. This enables fast and simple computation of the deformed image for any scan that lives in the same real-world coordinates as the corresponding input.

### Implementation details

The generative model has a number of parameters, which we take directly from^26^. We note that this choice of parameters generates MRI contrasts, resolutions, and bias fields that go well beyond what one realistically encounters in real data; such aggressive simulations enables the CNN to generalize better in practice.

The architecture of the registration CNN is the same as the in the model distributed with SynthMorph^24^, which is, to the best of our knowledge, the only publicly available neural network for nonlinear registration across non-predefined contrasts. In short, this CNN is a 3D regression U-net^58^ with four resolution levels in the encoder and three in the decoder. Each encoder block has a stride-2 convolution and a LeakyReLU layer (parameter 0.2). Each decoder block has a stride-1 convolution, an upsampling layer, and a skip connection to the corresponding block in the encoder. The last layer of the decoder is followed by three further convolutional blocks and an upsampling layer that brings the size back to the original resolution. All convolutions have size 3 × 3 × 3. The number of features is 256 for all layers.

For training, we finetuned the SynthMorph model (with the weights of the last layer divided by 2, as explained in Section “Training”) for 100,000 iterations using the Adam optimizer^59^ and a learning rate of $10^{-4}$. We used the same trade-off parameter as in the original paper: $\lambda =1.0$. The networks are implemented in TensorFlow/Keras^60,61^. Crucially, the inference code is implemented in a version of Python that is distributed with FreeSurfer, such that users do not need to create virtual environments and install dependencies.

We also emphasize that all the components of the pipeline are differentiable: the segmentation, the computation of centroids, the affine alignment, and the nonlinear registration. Therefore, *EasyReg* could be used as a building block in meta-architectures that include registration components.

## Experiments and results

### MRI data

We performed experiments using seven different datasets:

- **T1**: 100 1 mm isotropic T1 scans, randomly selected from the publicly available IXI dataset (www.brain-development.org/ixi-dataset/).
- **T1b**: to test the registrations across T1 scans acquired with different sequences, we also used 100 1 mm isotropic T1 scans from the publicly available MindBoggle dataset^62^.
- **T2**: 100 1 mm isotropic T2 scans randomly selected from IXI (leaving out the subjects corresponding to the 100 T1 scans).
- **FA**: 100 fractional anisotropy (FA) volumes derived from the diffusion MRI data from 100 subjects randomly selected from the HCP dataset (leaving out the subjects whose data were used in training). These FA volumes have 1.25 mm isotropic resolution.
- **AxFLAIR**: 100 FLAIR scans with 1$\times$1$\times$5 mm axial resolution (5 mm slice thickness), from 100 randomly selected subjects from the ADNI dataset (leaving out the subjects whose data were used in training).
- **SagT1**: We downsampled the 100 T1 scans to 1$\times$1$\times$5 mm sagittal resolution (5 mm slice thickness).
- **AxT2**: We downsampled the 100 T2 scans to 1$\times$1$\times$5 mm axial resolution (5 mm slice thickness).

Ground truth segmentations at 1 mm isotropic resolution were obtained for every dataset using the method implemented in FreeSurfer 7.3.0^63^. For T1 and T1b, FreeSurfer was run directly on the scans. For the rest of datasets, FreeSurfer was run on the companion 1 mm T1 scans that are available for IXI, HCP, and ADNI, after registration to the T2/FLAIR/diffusion scans. We note that running FreeSurfer after registration introduces a slight amount of blurring but avoids nearest neighbor interpolation artifacts associated with deforming segmentations.

### Registration experiments

In order to evaluate different aspects of the registration method, we used eight different experimental setups, each comprising 100 registrations:

- **T1–T1**: We first registered each 1 mm T1 scan to another 1 mm T1 scan from the same dataset. This is a frequent scenario in neuroimaging—and also the setup in which classical techniques best perform.
- **T1–T1b**: We register across the two different T1 datasets with 1 mm resolution, in order to assess the impact of differences in acquisition platform and parameters.
- **T1–T2**: Registering T1 and T2 scans at 1 mm resolution enables us to assess the impact of registering across modalities, while keeping the resolution high and isotropic.
- **T1–FA**: This setup enables us to assess the accuracy when registering across modalities with inherently different mechanisms to generate image contrast (and slightly different resolution).
- **T1–AxFLAIR**: We now consider registration between 1 mm T1s and clinical scans with large slice spacing (5 mm) and different contrast (FLAIR).
- **SagT1–SagT1**: We use this setup to assess the impact of large slice spacing, while sharing the same MRI modality and resolution.
- **AxT2–AxFLAIR**: This is similar to the previous setup, but using images with different MRI modality (T2 and FLAIR).
- **SagT1–AxFLAIR**: The most general case, where the images have different MRI modality (T1 vs FLAIR), large slice spacing, and different orientation (sagittal vs axial).

### Competing methods

We used the following competing methods in our experiments:

- **NiftyRegAffine**: the block matching method implemented in NiftyReg^53^, with default parameters.
- **MI-Affine**: a linear registration method based on mutual information^64^, which is widely used in neuroimaging (e.g., it is implemented in SPM and FreeSurfer). We used the implementation in FreeSurfer (“mri_coreg”).
- **EasyRegAffine**: the affine component of our proposed method, used in isolation.
- **NiftyReg**: the full NiftyReg pipeline, with its linear and nonlinear modules. For the nonlinear algorithm, we used the diffeomorphic mode (based on SVFs) with control point spacing of 5 mm when both input images are isotropic, and 20 mm if at least one of them has large slice spacing. As cost functions, we used local normalized cross correlation within modalities (radius: 4 mm) and mutual information across modalities. These parameters were set based on pilot experiments on withheld scans from the different datasets.
- **ANTs**: the full ANTs pipeline with affine and nonlinear “SyN” models. For isotropic inputs, we used a smoothing factor $\sigma =3$; for large slice spacing, we use $\sigma =6$. Within modalities, we used the local normalized cross correlation (semi-radius: 4 mm, as for NiftyReg); across modalities we used mutual information. As for NiftyReg, these parameters were set based on pilot experiments on withheld scans from the different datasets.
- **SynthMorph**: we used the publicly available implementation of SynthMorph, which requires affine registration to a reference space; we used “mri_coreg” for this purpose.
- ***EasyReg***: the proposed method.

We note that SynthMorph is, to the best of our knowledge, the only deep learning registration method that can align two scans without knowing their contrast a priori. This is the reason why it is the only learning-based competing method in our experiments. We also note that the classical methods benefit from skull stripping; we used SynthStrip^52^ for this purpose. Likewise, SynthMorph requires skull stripping, for which we also use SynthStrip. We note than our proposed method does not require skull stripped inputs.

### Accuracy metrics

As is commonplace in the registration literature, we use segmentation quality metrics as a proxy for registration accuracy. Evaluating registration accuracy directly would require ground truth correspondences that are very difficult to obtain, particularly across subjects. Instead, we register a scan to another, use the resulting transform to deform the labels, and compare these deformed labels with the labels of the fixed image in native space. We use three different metrics to quantify the agreement of label maps: Dice overlap^65^, the mean distance between the surfaces of the two segmentations, and the 95th percentile of the distribution of the surface-to-surface distance (henceforth, the “95% Hausdorff distance”). This 95th percentile is a robust version of the Hausdorff distance, i.e., the maximum distance between the surfaces. All the metrics were averaged across 29 ROIs defined by FreeSurfer: the 27 subcortical ROIs, the left cerebral cortex, and the right cerebral cortex.

Even though all the competing methods are diffeomorphic in their continuous formulation, their discrete implementations lead to negative Jacobian determinants in practice. Therefore, we also count the number of voxels for which the Jacobian determinant is negative; i.e., the voxels where the deformation field folds onto itself and the one-to-one mapping is lost. This quantity enables us to compare the regularity and invertibility of the deformation fields produced by different methods.

Finally, we also report the inverse consistency error for the different methods:

$$\begin{aligned} ICE = \frac{1}{2|\Omega |} \sum _{\varvec{x}\in \Omega } \Vert \varvec{x} - \hat{\varvec{T}^{-1}}(\hat{\varvec{T}}(\varvec{x})) \Vert + \frac{1}{2|\Omega |} \sum _{\varvec{x}\in \Omega } \Vert \varvec{x} - \hat{\varvec{T}}(\hat{\varvec{T}^{-1}}(\varvec{x})) \Vert . \end{aligned}$$

We note that this error will be greater than zero for the symmetric methods, due to the discrete integration. Importantly, we also note than SynthMorph is *not* inverse consistent, so the error is expected to be much higher than for the other approaches.

**Figure 3:**
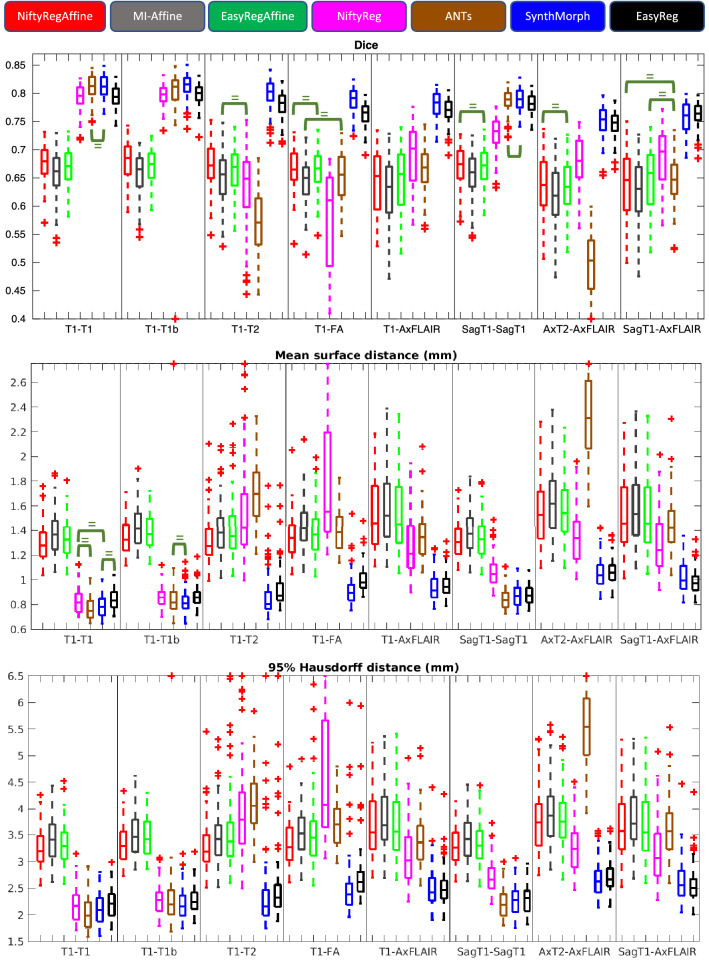
Box plot for the registration accuracy metrics achieved by the different methods in the different registrations tasks: Dice scores (top), mean surface-to-surface distances (center), and 95% Hausdorff distance (bottom). On each box, the central mark is the median, the edges of the box are the 25th and 75th percentiles, and the whiskers extend to the most extreme datapoints that are not outliers (which are plotted as crosses). The green brackets indicate *lack* of statistically significant differences between two methods ($p>0.05$), according to a non-parametric Wilcoxon test; note that we mark the lack (rather than existence) of differences not to clutter the figures, since most differences are significant (due to the large sample size and to the fact that the values have little noise, as they are averages over brain structures).

**Figure 4:**
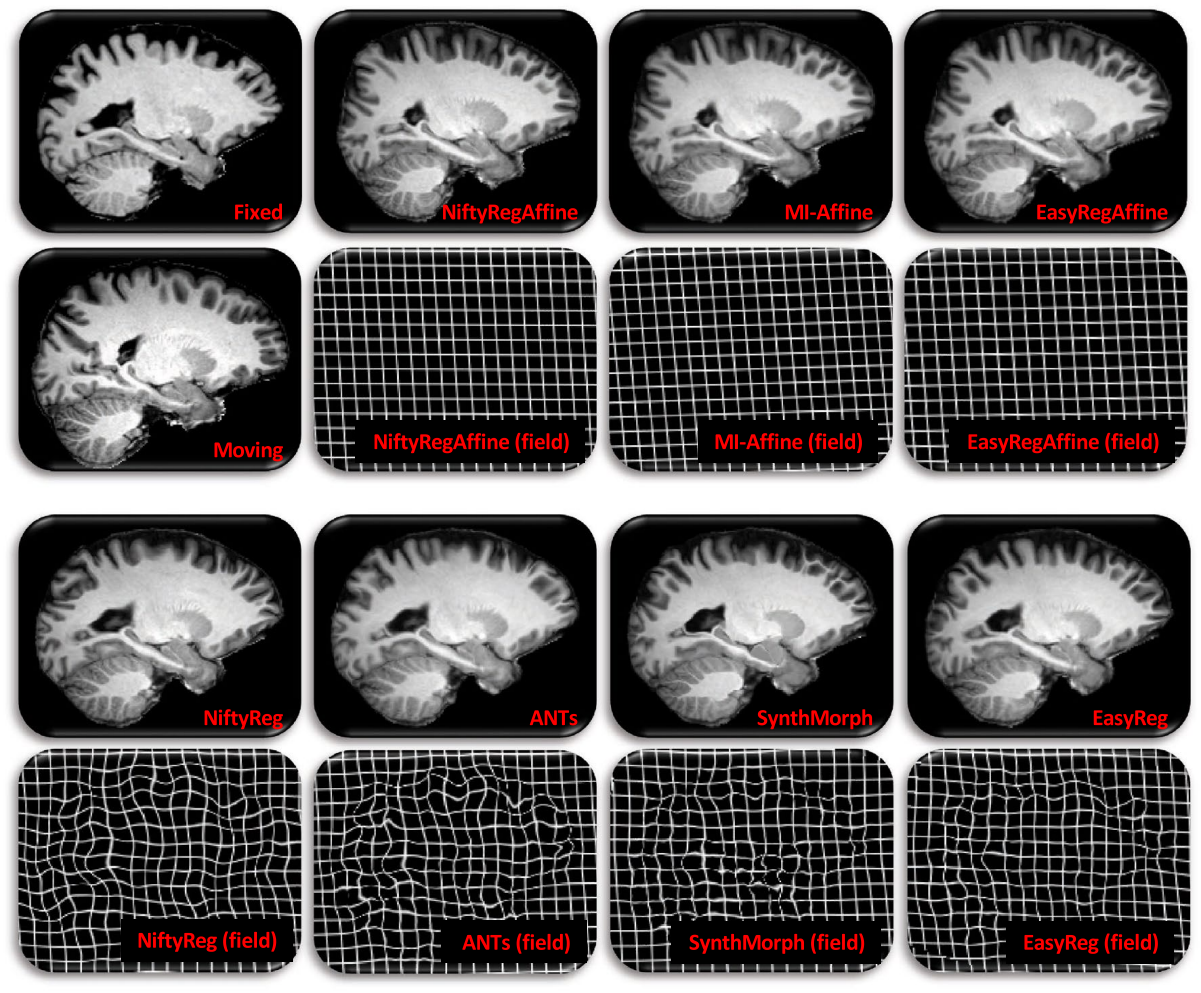
Sample registration from T1–T1b setup, i.e., MindBoggle to IXI Top left: sagittal slices of fixed and moving images. Rest: corresponding registered slice and deformation field (represented as a deformed grid) for the seven competing methods.

**Figure 5:**
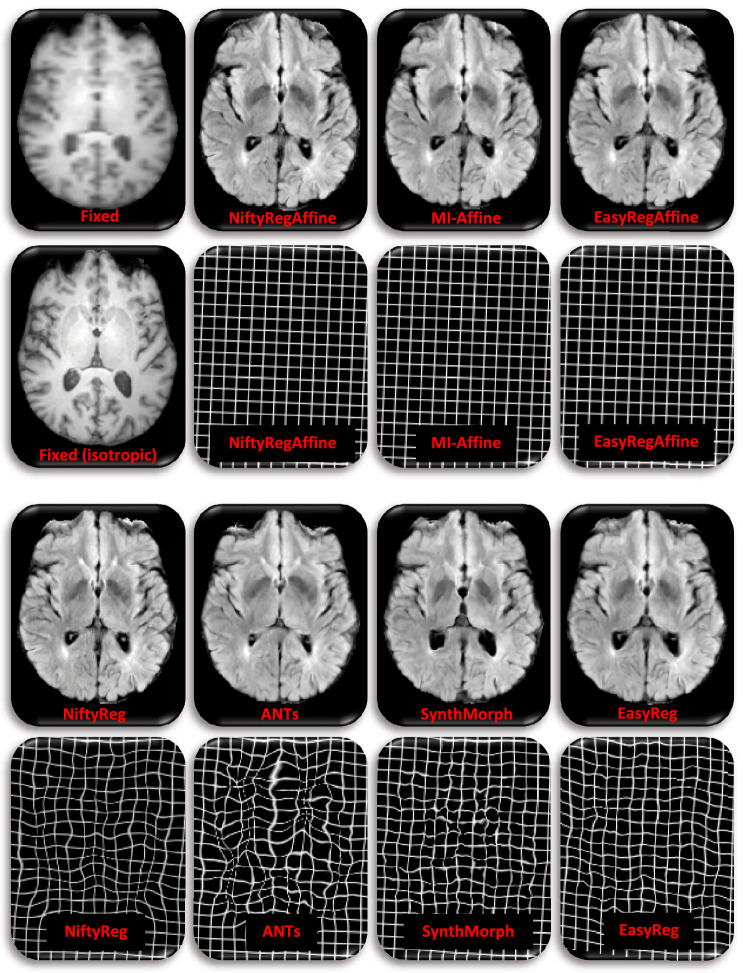
Sample registration from SagT1–AxFLAIR setup, i.e., 5 mm axial FLAIR from ADNI, to 5 mm sagittal T1-weighted scan from IXI. Top left: axial slice of the fixed image and corresponding isotropic slice; the later was *not* used in registration; we display it for easier qualitative assessment of registration accuracy. Rest: corresponding registered slice and deformation field (represented as a deformed grid) for the seven competing methods.

### Results

Figure 3 shows box plots for the Dice overlap, mean surface distance and Hausdorff distance, for all the registration experiments and competing methods—as well as the results of non-parametric Wilcoxon tests comparing the medians of the different methods. Tables 1 and 2 show the number of voxels with negative Jacobian determinants and the average inverse consistence error, respectively. Figures 4 and 5 show registration examples for an intra-modality (T1-T1b) and an inter-modality setup (SagT1-AxFLAIR).

**Table 1:** Number of voxels with negative Jacobian determinants, for the different methods and registration tasks.

Task	NiftyReg	ANTs	SynthMorph	EasyReg
T1–T1	0.09	0.02	0.91	< 0.01
T1–T1b	0.12	0.04	1.12	< 0.01
T1–T2	0.10	0.03	1.10	< 0.01
T1–FA	0.14	0.09	1.21	< 0.01
T1–AxFLAIR	0.23	0.11	0.97	< 0.01
SagT1–SagT1	0.27	0.08	0.26	< 0.01
AxT2–AxFLAIR	1.45	0.53	88.58	46.22
SagT1–AxFLAIR	0.54	0.26	0.25	< 0.01

Only voxels inside the brain are considered.

**Table 2:** Average inverse consistency errors (in mm), for the different methods and registration tasks.

Task	NiftyReg	ANTs	SynthMorph	EasyReg
T1–T1	0.02	0.01	1.60	0.08
T1–T1b	0.02	0.02	1.69	0.08
T1–T2	0.02	0.01	1.53	0.06
T1–FA	0.03	0.02	1.61	0.07
T1–AxFLAIR	0.04	0.03	1.73	0.08
SagT1–SagT1	0.04	0.02	1.69	0.08
AxT2–AxFLAIR	0.06	0.04	1.80	0.07
SagT1–AxFLAIR	0.04	0.02	1.66	0.08

Errors are averaged over voxels inside the brain.

### Affine registration

The results for NiftyReg (affine) and the mutual information method are very similar to each other. Moreover, they remain fairly constant across metrics and registration experiments. For example, the median Dice scores are all between 0.62 and 0.68—which is very little variation, considering the range of MRI modalities, orientations, and resolutions involved in the experiments. This is because, given skull-stripped scans, both methods are quite robust (even though NiftyReg is often 1–2 Dice points and 0.1–0.2 mm better than the mutual information method).

Our proposed affine algorithm produces approximately the same results as the other two affine approaches: the boxes corresponding to our method in Fig. 3 are most often between those of the other two affine techniques. Moreover, there are not noticeably more outliers for any of the three affine registration techniques. We can thus conclude that the proposed affine module, which does not require any preprocessing, is as accurate and robust as widespread affine registration techniques.

### Nonlinear registration

The box plots in Fig. 3 and the example in Fig. 4 show that, when registering 1 mm isotropic T1 scans to each other (even across datasets), all methods (classical and learning-based) are very accurate: Dice scores range between 0.79 and 0.81, mean surfaces distances between 1.3 and 1.4 mm, and 95% Hausdorff distances between 3.2 and 3.5 mm. However, when the modalities or resolutions of the input scans differ, the performance of the classical methods quickly drops, whereas the learning-based techniques yield Dice scores over 0.75 across the board, including the most challenging setups (e.g., when registering scans of different MRI modalities with large slice spacing). The same trend is observed for the surface distances. Qualitatevely speaking, the example in Fig. 5 shows how classical techniques fail to align even high-contrast regions, like the lateral ventricles—which are well registered by the learning-based methods.

Compared with SynthMorph, our proposed approach loses 1–2 Dice points and 0.1–0.2 mm in some of the setups; this is the price to pay for the smoother, symmetric fields. For example, the field produced by SynthMorph in the intra-modality registration example in Fig. 4 is highly convoluted, with plenty of deformation in flat regions (not penalized by segmentation metrics) that lead to a cartoon-like deformed image. *EasyReg*, on the other hand, yields a smooth field, which is very similar to those produced by NiftyReg and ANTs (which are remarkably similar to each other). The same effect is observed across modalities in Fig. 5, where SynthMorph creates strong local variations in the deformation field that are not realistic, compared with the much smoother fields produced by *EasyReg*—which, in the setup of the example (SagT1–AxFLAIR), yields the same median Dice score and surface distances (Fig. 3).

The regularity of the deformation fields is also reflected in the number of voxels with negative Jacobians (Table 1). All the approaches produce highly regular fields, with less than one “flipped” voxel in almost every setup. The only exception occurs when the aial T2s and axial FLAIRs are registered with the deep learning approaches, which leads to 88 (SynthMorph) and 46 (*EasyReg*) flipped voxels per scan; we note that this is a tiny fraction (<0.01%) of the total number of voxels. Outside that registration task, *EasyReg* yields less than 0.01 flipped voxels per scan all across the board.

Finally, Table 2 shows the average inverse consistency errors for the different methods and registration tasks. We note that SynthMorph is not an inverse consistent approach, so it yields fairly large consistency errors across the boards, almost 2 mm on average. NiftyReg, ANTs, and *EasyReg*, on the other hand, yield very small averages (below 0.1 mm) for all registration tasks. We note that EasyReg produces slightly larger errors than the classical approaches, despite using the same integration method as NiftyReg. This is for two reasons: the fact that our CNN uses single (rather than double) floating-point precision, and the fact that NiftyReg uses an adaptive strategy for the number of integration steps—which is constant in our CNN.

## Discussion and conclusion

In this article, we have presented *EasyReg*, a learning-based registration method for brain MRI that is: symmetric; modality- and resolution-agnostic; diffeomorphic; affine and nonlinear; and parameter-free. *EasyReg* requires no preprocessing and can be used with unpreprocessed data direct from the scanner. *EasyReg* is publicly available as part of FreeSurfer and can be used out of the box, without dedicated hardware or machine learning expertise.

*EasyReg*’s run time on a modern desktop without a GPU is approximately 2 mins—or just one minute, if the input scans have already been registered to other images and the computation of the centroids can be bypassed. This is in contrast with classical techniques: ANTs ran on approximately 35 mins on the same computer, while NiftyReg ran in 20 mins. SynthMorph runs in approximately 30 s, since it only does one forward pass through the network (*EasyReg* does two passes; see Eq. (2)).

Our experiments covered a wide range of setups, demonstrating *EasyReg*’s ability to cope with large variations in scan orientation, resolution, and pulse sequence. This is in contrast with classical techniques, which cannot handle such dissimilitudes. Compared with SynthMorph, on which it builds, *EasyReg* is symmetric (up to numerical precision of the SVF integration), produces smoother deformation fields, and does not require preprocessing (skull stripping, registration to a template). *EasyReg*’s symmetry comes at the expense of a minimal loss of accuracy around label boundaries. Therefore, SynthMorph may be more appropriate for inherently asymmetric tasks where field irregularity is irrelevant (e.g., segmentation), while *EasyReg* is a better choice when robustness or minimization of directional bias are the priority.

*EasyReg* is independent of resolution, as the deformations fields are always computed on a 1 mm isotropic grid—which is the resolution to which the inputs of the CNN are resampled. Therefore, *EasyReg* implicitly learns to super-resolve scans of lower resolution to 1 mm isotropic. However, if the resolution is higher than that, *EasyReg* still operates at 1 mm internally and cannot exploit the higher resolution. Capitalizing on such smaller voxel sizes requires a training dataset with higher resolution and remains as future work.

The proposed framework is also independent of MR contrast (i.e., pulse sequence), but relies on an image formation model that is fairly specific to MRI, including artifacts like bias field. While this approach may cope with other modalities that may be modeled by a Gaussian mixture (e.g., computerized tomography^26^), a different image formation model would be required to handle other modalities like ultrasound or positron emission tomography (PET).

A limitation of our experimental setup is that we did not exhaustively sweep all the parameters of the classical methods using the full datasets, which would have been computationally very expensive. While the default settings produce state-of-the-art results in the intra-modality setups, it is very possible that exhaustive finetuning of the parameters for every scenario (or even every case separately) may yield better results. For example, the NiftyReg parameters we used across modalities worked well for T1–AxFLAIR, but poorly for T1–FA.

*EasyReg*, on the other hand, has no parameters. While this is an advantage in terms of simplicity and reproducibility, it is also possible that the ability to tune the trade-off parameter $\lambda$ may enable the user to improve the results, e.g., by increasing $\lambda$ in inter-modality scenarios. While frequent retraining for different values of $\lambda$ is impractical, recent advances could enable training of a single $\lambda$-adaptive network, such that the user can specify the value of this parameter for each scan at test time. Examples of such approaches include Hypermorph^66^, where the weights of the CNN are given by a separate network that takes $\lambda$ as input, or LapIRN^67^, which modulates the feature statistics of the deeper layers instead (and is thus much less memory intensive). Exploring this direction, along with other architectural improvements that may have a positive impact on *EasyReg* (e.g., progressive deformations^68^), remains as future work.

By enabling fast and symmetric registration of unpreprocessed brain MRI scans, *EasyReg* holds great promise to ease the adoption of learning-based registration method by neuroimaging pipelines.

## Data Availability

All the brain MRI scans used in this article were obtained from publicly available datasets: HCP, ADNI, IXI, and MindBoggle: HCP: https://www.humanconnectome.org/study/hcp-young-adult/data-releases. ADNI: https://adni.loni.usc.edu/. IXI: https://brain-development.org/ixi-dataset/. MindBoggle: https://mindboggle.info/. We also note that the ADNI Data Use Agreement (available at https://ida.loni.usc.edu/collaboration/access/appLicense.jsp) requires that we note that: “Data used in preparation of this article were obtained from the Alzheimer’s Disease Neuroimaging Initiative (ADNI) database (http://adni.loni.usc.edu). As such, the investigators within the ADNI contributed to the design and implementation of ADNI and/or provided data but did not participate in analysis or writing of this report. A complete listing of ADNI investigators can be found at: adni.loni.usc.edu/wp-content/uploads/how_to_apply/ADNI_Acknowledgement_List.pdf”. The ADNI Data Use Agreement also requires us to note that: “The ADNI was launched in 2003 as a public-private partnership, led by Principal Investigator Michael W. Weiner, MD. The primary goal of ADNI has been to test whether serial magnetic resonance imaging (MRI), positron emission tomography (PET), other biological markers, and clinical and neuropsychological assessment can be combined to measure the progression of mild cognitive impairment (MCI) and early Alzheimer’s disease”.
